# Microbial biosurfactant-mediated green synthesis of zinc oxide nanoparticles (ZnO NPs) and exploring their role in enhancing chickpea and rice seed germination

**DOI:** 10.1186/s11671-024-04134-1

**Published:** 2024-11-01

**Authors:** Indukalpa Das, Debajit Borah

**Affiliations:** Department of Biotechnology, The Assam Royal Global University, Guwahati, 781035 India

**Keywords:** Metal nanoparticles, Green synthesis, Agricultural application, Seed germination, Bacterial biosurfactant

## Abstract

Malnutrition is one of the greatest challenges faced by humanity, which may be addressed by improving crop productivity to ensure food security. However, extensive use of synthetic fertilizers can lead to soil fertility degradation. This study highlights the potential of combining nanotechnology with biotechnology to enhance the germination rates of commercially important crop seeds. Bacterial biosurfactant extracted from a newly isolated *Klebsiella* sp. strain RGUDBI03 was used as a reducing and capping agent for the synthesis of zinc oxide nanoparticles (ZnO NPs) through a simple method. Extensive characterization of ZnO NPs through electron microscopic analysis showed well-dispersed, homogeneous NPs with a size range of 2–10 nm. High-resolution transmission electron microscopy (HR-TEM) images also revealed molecular fringes of 0.26 nm in single crystal ZnO NPs, with approximately 50% of the NPs exhibiting a size range of 2–4 nm. X-ray diffraction (XRD) results of ZnO NPs indicated the presence of (100), (002), (101), (102), (200), and (112) planes, confirming their crystalline nature. The presence of C = C–H, C = C, C–H, and C = C groups in both the bacterial biosurfactant and ZnO NPs, as depicted by Fourier-transform infrared spectroscopy (FTIR) spectra, confirmed the function of the biosurfactant as a reducing and capping agent. The nano-primed chickpea (*Cicer arietinum*) and rice (*Oryza sativa*) seeds showed an increase in water uptake rate, 89% and 92% respectively, compared to the control (73% and 44%), leading to an enhanced germination rate of 98% and 76%, compared to their respective controls (80% and 30%) under optimized conditions. Additionally, the nano-primed seeds exhibited higher levels of α-amylase activity in both seeds (0.37 mg/g for chickpea and 2.49 mg/g for rice) compared to the control. Notably, the ZnO NP priming solution exhibited no cytotoxicity on red blood cells and earthworms (*Eudrilus eugeniae*), indicating their non-cytotoxic and eco-friendly nature for future field trials.

## Introduction

Global hunger has been increasing significantly in recent years. In 2016, 11% of the world's population was undernourished, driven by the growing global population, projected to reach 9.8 billion by 2050 [1, 2]. According to the FAO, an estimated 821 million people worldwide were undernourished in 2017, accounting for nearly one in nine people [3]. Food insecurity poses a serious threat to achieving the Sustainable Development Goal (SDG) of eradicating hunger [4]. The global population is projected to grow steadily from 7 to 10 billion by 2050, with an annual increase of 70 million individuals [5]. This growth will result in a 70% increase in food production demand [2, 6–8]. Therefore, enhancing agricultural output and ensuring food sufficiency through efficient fertilizer use is crucial, especially given the limited availability of arable land [9].

In traditional farming, chemical fertilizers are commonly used to boost crop production. However, plants can directly utilize only 10–40% of the applied fertilizers [10]. The residual fertilizers either remain in the soil as insoluble inorganic salts or are washed into nearby rivers, posing a significant risk to global soil biodiversity [10].

Nanoscience has been growing at an increasing rate with innumerable advancements in various fields due to its unique structural properties [11]. Scientists have recently started exploring the use of nanomaterials in agricultural applications, which requires more attention and research data. Hence it is believed that the integration of nanotechnology and biology may solve a wide range of problems and bring about a remarkable change in the agriculture sector [12].

The global nano-fertilizer market is divided into North America, Europe, Asia Pacific, Latin America, and the Middle East [13, 14]. In 2022, North America held the largest market share, contributing over 34% of the revenue [14]. Countries like the US have transitioned from conventional fertilizers to nano-fertilizers to enhance crop productivity [13]. Recently, the Indian Farmers Fertilizer Cooperative (IFFCO) developed and patented nano-urea in liquid form (Indian patent number 400681) [15]. This innovation, an alternative to commercial urea, is being exported to countries including Brazil, South Africa, and Sri Lanka [15]. Currently, IFFCO is the sole global producer of nano-urea. The global nano-urea market is segmented by end-user industries, including food crops, cash crops, plantation crops, horticulture crops, and others. The food crops category dominated the market in 2023, accounting for almost 50%, and is expected to continue its strong performance through 2035 [13].

However, there is very limited understanding on the ultimate fate of nanoparticles (NPs) in both the environment and living organisms. Only a few studies have examined the possible drawbacks of the use of NPs, including the risks it poses to farm workers and the potential toxicity it may have on the health of consumers of smart agricultural products [16]. Additionally, the production of NPs using traditional methods involves costly chemical and physical procedures that carry the risk of causing ecological harm, cellular toxicity, and carcinogenic effects. These issues occur as a result of employing hazardous substances including organic solvents, reducing agents, and stabilizers to prevent excessive agglomeration of the colloids [17].Therefore, it is imperative to develop NPs using environmentally friendly methods, utilizing them as a priming solution for seedlings rather than as soil fertilizers to minimize NP usage and reduce the risk of soil toxicity from long-term use.

Phyto-synthesis has emerged as a promising and environmentally friendly substitute for traditional physical and chemical techniques, receiving significant interest from researchers [18]. However, the extensive use of plant and animal extracts may pose a threat to biodiversity. The use of microbial biosurfactants to synthesize NPs has gained popularity in research applications due to its eco-friendly nature and capacity to generate cost-effective NPs without harming biodiversity [19].

Nanoparticles have been recently employed as a potential seed growth enhancer, as they have the capacity to serve as a fertilizer, pesticide, and nutrient for seed growth, in addition to providing effective seed dormancy [20]. Researchers have demonstrated various experiments on the beneficial impact of NPs in agriculture and environmental applications, particularly in enhancing seed quality parameters by increasing water absorption and enzymatic activity in seeds, as well as increasing their defense mechanisms against pathogens and pollutants [20]. Studies have shown that ZnO NPs can increase agricultural yield by enhancing seed germination rates, sugar levels, and antioxidant activity [21]. The ZnO NPs also serve as powerful antibacterial agents due to their small size (less than 100 nm) and extensive surface area [22]. Thus, ZnO NPs are of interest in both medicinal research and the food sector, although their role in seed germination has received less attention compared to other areas [23, 24]

Several species of the *Klebsiella* genus are recognized for their capacity to break down hydrocarbons through biosurfactant production, but their potential for NP synthesis has not been thoroughly investigated [25, 26]. A newly isolated *Klebsiella* sp. strain RGUDBI03, capable of producing biosurfactants, has demonstrated potential in reducing silver salts into silver nanoparticles (Ag NPs) [27]. While Ag NPs have been studied for their role in seed germination [28], their potential cytotoxicity could limit their field application [29]. Zinc salts, widely used in agriculture as essential micronutrients to promote plant growth, are considered safe by many researchers [30, 31]. Thus, reducing zinc salts into ZnO NPs using bacterial surfactants may enhance their efficacy in promoting the germination rate of cash crops such as rice, maize, and wheat.

Rice is a common cash crop and a staple food in most Southeast Asian countries, including India, with a global market valued at USD 287.45 billion in 2021[32, 33]. Additionally, chickpea (*Cicer arietinum*), a significant pulse crop in India, is widely consumed around the world [34]. Therefore, in order to mitigate the issue of global food hunger, it is essential to promote the use of modern bio-synthesized nanomaterials in agricultural fields. This paper focuses on the effect of biosurfactant-mediated ZnO NPs to enhance seed germination, providing a route to encourage smart agricultural practices.

## Materials and methods

### Preliminary qualitative assessment of the biosurfactant producing *Klebsiella* sp. strain RGUDBI03

The previously isolated biosurfactant-producing *Klebsiella* sp. strain RGUDBI03 (GenBank accession no. ON945613.1) was obtained from the repository of the Department of Biotechnology at The Assam Royal Global University, Guwahati. The strain was revived in nutrient broth and maintained at 37 °C and 135 rpm in a rotary shaker (ORBITEK®-LE, Scigenics Biotech India Pvt. Ltd) for 24 h. It was then cultured on nutrient agar plates and stored for future use.

*Klebsiella* sp. strain RGUDBI03 was inoculated in BH broth in the presence of 2% (*v/v*) diesel oil and maintained at 37 °C and 135 rpm in a rotary shaker for 6 days. The cell-free medium was obtained by collecting the supernatant after centrifuging the cells at 10,000 rpm for 10 min at 4 °C. This cell-free medium was used for the qualitative assessment of the biosurfactant.

#### Drop collapse assay

The cell-free extract was initially analyzed for its ability to reduce the surface tension of the diesel oil-supplemented medium using the drop-collapse method. A drop of the cell-free extract from the diesel oil-supplemented medium was placed onto a hydrophobic surface. The presence of biosurfactant in the cell-free extract was assessed based on its ability to destabilize the drop within 1 min [35]. Drops containing biosurfactant collapse, whereas drops of non-surfactant compounds remain stable [35].

### Determination of the emulsification index of the cell-free extract

The emulsification index of the bacterial culture was determined by rigorously vortexing 2.5 mL of diesel with an equal volume of bacterial cell-free extract for 60 s, followed by incubation at 37 °C for 24 h. The emulsification index (*E*_*24*_) was calculated using the following equation [36]:

$$Emulsification\; Index (E24)= \frac{Height\; of\; the\; emulsion\; layer \left(mm\right)}{Total\; height\; of\; the\; solution}\;\times\;100$$

### Production and extraction of biosurfactant from *Klebsiella* sp. strain RGUDBI03

The cell-free medium obtained from the culture was used for the extraction of crude biosurfactant using a pre-standardized cold acetone precipitation technique [37]. Briefly, the cell-free extract was combined with an equal volume of cold acetone and left to stir continuously overnight. The presence of white powdery particles following intense stirring with chilled acetone indicated the formation of a biosurfactant precipitate. The layer of biosurfactant that formed at the bottom was collected by centrifuging at 10,000 rpm for 10 min under ice-cold conditions (NEYA 16R cooling centrifuge, REMI India Pvt. Ltd). The resulting solid mass was then obtained by air drying [38].

### Characterization of the crude biosurfactant

The crude biosurfactant sample was then subjected to FTIR spectrometric analysis within the range of 4000–500 cm^−1^ to determine the presence of various functional groups. The sample was prepared by grinding about 5–10 mg of crude biosurfactant with KBr [27]. The biosurfactant and KBr mixture was then formed into a pellet, which was used for FTIR analysis [39].

### Synthesis and characterization of ZnO NPs

The ZnO NPs were produced by reducing 10 mL of 1 mM ZnCl_2_ with 50 mg of bacterial biosurfactant in an autoclave maintained at 121 °C and 15 psi for 15 min (Equitron Vertical Autoclave Sledd 7411–5576, Equitron India Pvt. Ltd.). The solution was then cooled to room temperature and centrifuged at 10,000 rpm for 10 min to allow the ZnO NPs to settle. The resulting pellet was desiccated into a fine powder using a hot air oven and preserved for further studies.

The ZnO NPs were analyzed and examined using FTIR (in the spectrum range of 4000–500 cm⁻^1^), SEM–EDX, HR-TEM (JEM-2100 Plus Electron Microscope, Japan Ltd.), and XRD (ULTIMA IV, Rigaku Japan Ltd.). The XRD results were compared with the standard powder diffraction card of JCPDS, zinc oxide card No. 36-1451. The thermal stability of the ZnO NPs was assessed using differential thermal analysis (DTA) and thermogravimetric analysis (TGA) (NETZSCH STA 44F3 GmbH instrument from Germany) within the temperature range of 28–600 °C.

### ZnO NPs mediated seed germination and plant growth assay

Fifty healthy chickpea seeds (*n* = 50) were surface-sterilized using 70% ethanol for 2 min, followed by washing with distilled water for the same duration. The sterilized seeds were then treated with different concentrations of ZnO NPs (10, 20, 30, and 40 mg/L) for 3 h. Seeds treated with distilled water and various doses of ZnCl_2_ (10, 20, 30, and 40 mg/L) served as control in the same experimental setup. After priming, the seeds were placed on moistened cotton beds and kept in dark conditions at room temperature for 3 days. The length of the seedlings was measured daily during this period [40].

Similarly, equal number of healthy rice seeds were prepared in the same manner, treated for 3 h, and then kept in darkness for 6 days. The treated seeds were subsequently examined for their water absorption ability, alpha-amylase activity levels, and overall soluble sugar content. Both the chickpea and rice seeds used in this study were procured from the local market located in Guwahati, Assam.

#### Seed water uptake assay

The amount of water absorbed by the seeds was determined by evaluating the change in weight of the seeds before and after priming. This change in weight was determined using the following formula [41]:

$$Water\; uptake\;(\%)=\frac{\text{Weight of seeds after priming}-\text{Weight of seeds before priming}}{\text{Weight of seeds before priming}} \times100$$

#### Assessment of germination percentage and the length of the germinated seedlings

The germination percentage of nano-primed chickpea and rice seeds was evaluated on the 3rd and 6th day after treatment, respectively. The determination was done using the following formula [42]:

$$Germination\; percentage\;(\%)=\frac{\text{Number of seeds germinated}}{\text{Number of seeds used}}\times100$$

The total length of the germinating chickpea seedlings was measured daily during the incubation period up to the third day. Similarly, the rice seeds that had been primed were examined periodically throughout the incubation period until the 6th day to measure the length of the germinated seedlings.

#### Alpha‑amylase activity assay

The concentration of α-amylase in the treated seeds was measured by determining the maltose content using dinitrosalicylic acid (DNS) reagent, following a standard method [40]. One gram of primed seeds was homogenized with 6 mL of chilled 1 M CaCl₂ solution at ice-cold temperatures, and then centrifuged for 25 min at 12,000 rpm to extract the α-amylase. A precise 100 µL of the enzyme extract supernatant was combined with 900 µL of distilled water and 1000 µL of 1% starch solution. The mixture was incubated at 37 °C for 15 min, followed by the addition of 1000 µL of DNS reagent. The solution was then maintained at 90 °C in a water bath for 5 min. After cooling, the solution was adjusted to a total volume of 10 mL by adding distilled water. The absorbance of the solution was measured at a wavelength of 540 nm [40]. The α-amylase activity was assessed by comparing the maltose concentration with a standard curve.

#### Total soluble sugar content

One gram of seeds was homogenized with 5 mL of 95% ethanol and centrifuged for 10 min at 10,000 rpm to determine the amount of soluble sugar (glucose) in the primed seeds [40]. About 5 mL of anthrone reagent was added to 1000 µL of the supernatant obtained from the centrifugation. After 17 min of incubation at 90 °C, the absorbance of the mixture was measured at 620 nm [40]. The data were compared to a standard glucose graph to determine the amount of soluble sugar in the primed seeds.

#### Cytotoxicity and soil toxicity assay of the synthesized NPs

A precise volume of 1000 µL of defibrinated sheep blood was obtained and centrifuged for 5 min at 4 °C and 1000 rpm. The pellet was then rinsed with 2 mL of PBS (phosphate-buffered saline) and subjected to centrifugation at 1000 rpm for 5 min at 4 °C. This rinsing process was repeated three times. The RBC sample mixture was prepared by combining 20 mL of PBS with the centrifuged pellet and carefully blending.

Specifically, 180 µL of the red blood cell (RBC) sample was incubated at 37 °C with 20 µL of various concentrations (10, 20, 30, and 40 mg/L) of ZnO NPs for 3 h. Following incubation, the tubes were cooled on ice for 5 min to halt the reaction, followed by 10 min of agitation. The mixture was then centrifuged at 2000 rpm for 5 min to ensure consistency. The percentage of hemolysis was determined by measuring the absorbance of 100 µL of the supernatant at 540 nm using a spectrophotometer at 4 °C. The degree of hemolysis was calculated using the following formula [43–45].

$$\text{Hemolysis }\left(\text{\%}\right)= \frac{\text{OD}540\text{ of Sample}-\text{OD}540\text{ of Test}}{\text{OD}540\text{ of positive control}-\text{OD}540\text{ of negative control}}\text{x }100$$

A group of twenty earthworms (*Eudrilus eugeniae*) were exposed to 10 mL of ZnO NPs at a concentration of 40 mg/L for 1 h. A control group was administered distilled water under identical conditions. Following the 1 h incubation period, each group of earthworms was transferred to an individual beaker along with its food source and monitored for 6 days. The treated earthworms were observed for any phenotypic alterations, including changes in color, behavior (geotaxis), and survival rate.

One earthworm from both the test and control groups was rinsed with a solution of 0.9% NaCl and 10% formalin. The gastrointestinal region was dissected using aseptic blades and preserved in 10% formalin for 24 h. The tissue material was thoroughly rinsed with running water for an extended period, and subsequently treated with various concentrations of alcohol (ranging from 10 to 100%) for 15 min each, with two changes. It was then cleaned with xylene for 10 min, also with two changes. The tissues were thereafter immersed in a solution of equal parts xylene and paraffin for 30 min in a water bath, followed by total immersion in paraffin for a minimum of 8 h [46].

Subsequently, tissues from each group were fixed using paraffin block preparation, and the resulting blocks were stored in water overnight. The blocks were then cut into thin slices using microtomy and treated with hematoxylin and eosin staining. Once the staining process was completed, the tissue slides were examined using a light microscope at a magnification of 100X [46].

### Statistical analysis

The studies were performed in triplicate, and the data are reported as the mean ± standard deviation (S.D.). A confidence level of 95% was used to evaluate statistical significance for *p*-values less than 0.05. A Student’s t-test was used to perform statistical comparisons between two means. The GraphPad™ online program was utilized for this purpose.

## Results

**Fig. 1:**
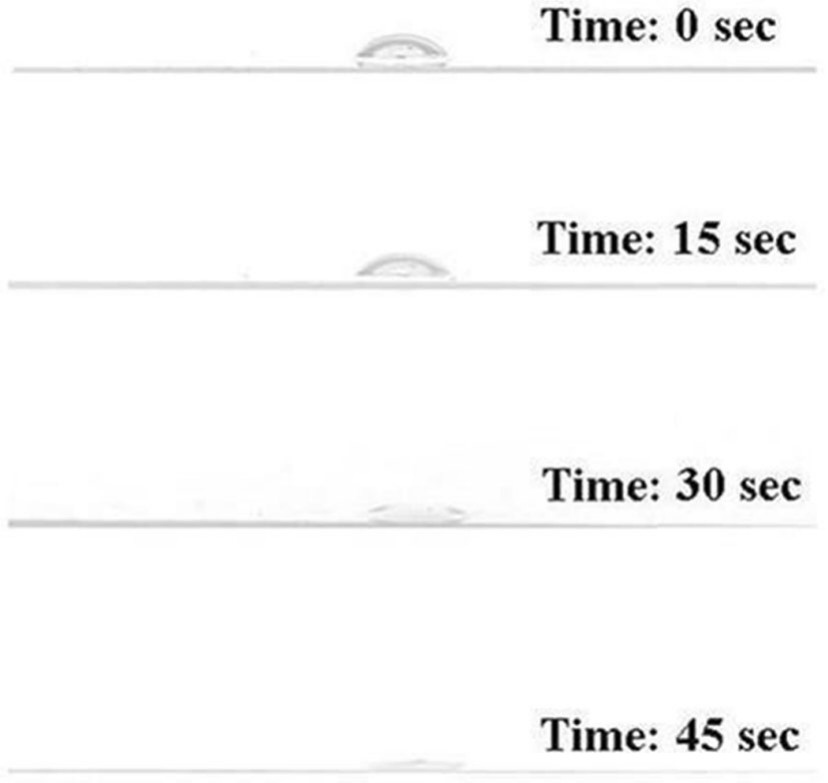
Drop collapse test result of the cell free extract of *Klebsiella* sp. RGUDBI03 at different time interval

**Fig. 2:**
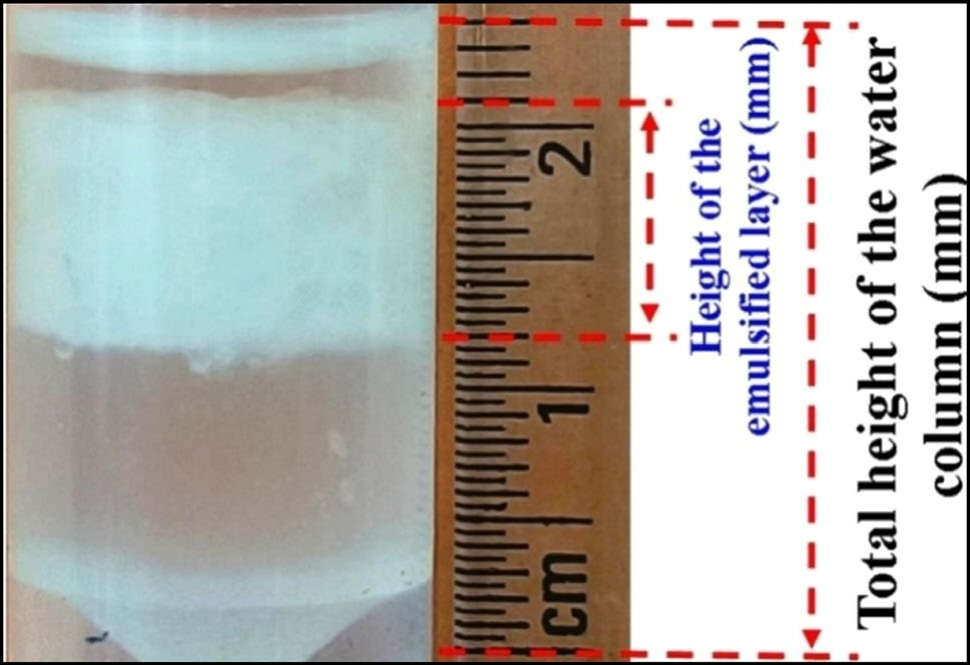
Result of emulsification index (*E*_*24*_) of the bacterial cell free extract with equal volume of diesel oil showing the height of the emulsified layer after 24 h of incubation

*Klebsiella* sp. RGUDBI03 (GenBank accession: ON945613.1) is a recently reported biosurfactant-producing Gram-negative, rod-shaped bacterial strain [27] which was revived to explore its ability to reduce ZnCl_2_ into stable ZnO NPs. The initial qualitative assessment of the cell-free extract of *Klebsiella* sp. RGUDBI03 demonstrated its ability to collapse a drop of diesel oil-supplemented media on a hydrophobic surface within 45 s, indicating its capacity to produce biosurfactant (Fig. 1). The emulsification index of the cell-free extract was checked after 24 h of incubation with an equal volume of diesel and was determined to be 35.71 ± 0.09% (Fig. 2).

FTIR spectroscopic analysis of the crude biosurfactant revealed clear peaks attributed to O–H (bend), O–H (stretch), CH_2_ (asymmetrical stretch), C = C (stretch), C–H (bend), C–H in-plane bends, and C = C–H (bends) as reported earlier (Table 1) [27].

**Table 1:** Interpretation of FTIR spectroscopy data of bacterial biosurfactant [27]

Sl. No	Frequency (cm^−1^)	Assignment
1	3454.78	O–H (bend)
2	3253.12	O–H(stretch)
3	2924.87	CH_2_ (asymmetrical stretch)
4	1621.72	C = C (stretch)
5	1376.37	C–H (bend)
6	1054.48	C–H in-plane bends
7	978.96	C = C–H (bends)

**Fig. 3:**
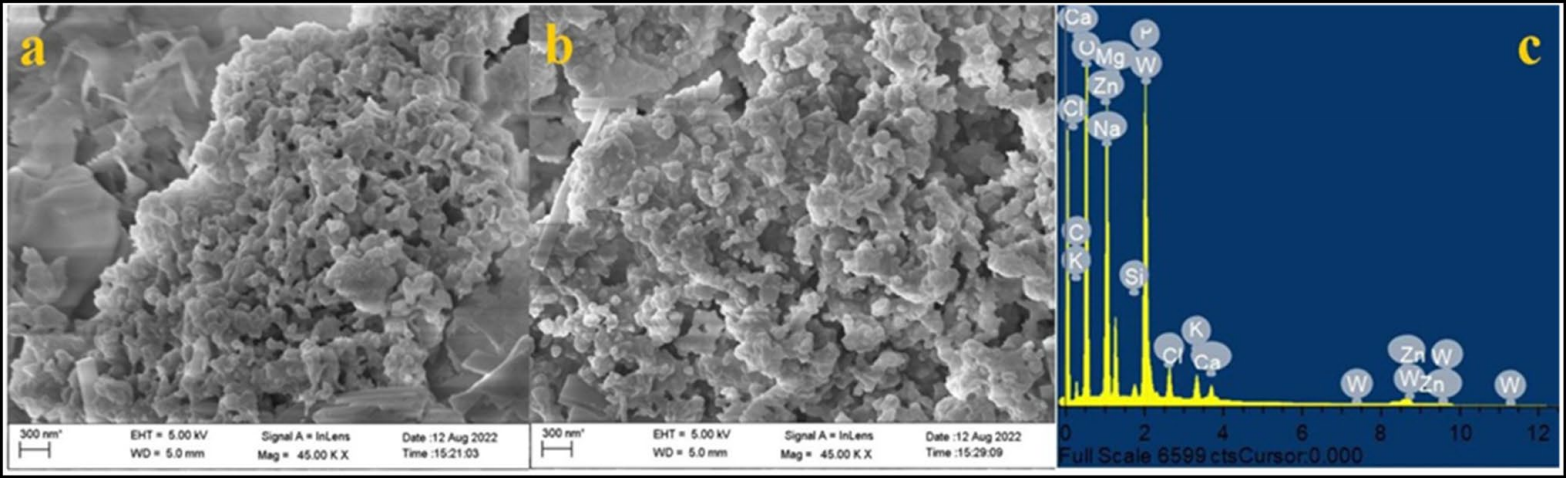
SEM images of **a**, **b** ZnO NPs and **c** EDX spectra

**Fig. 4:**
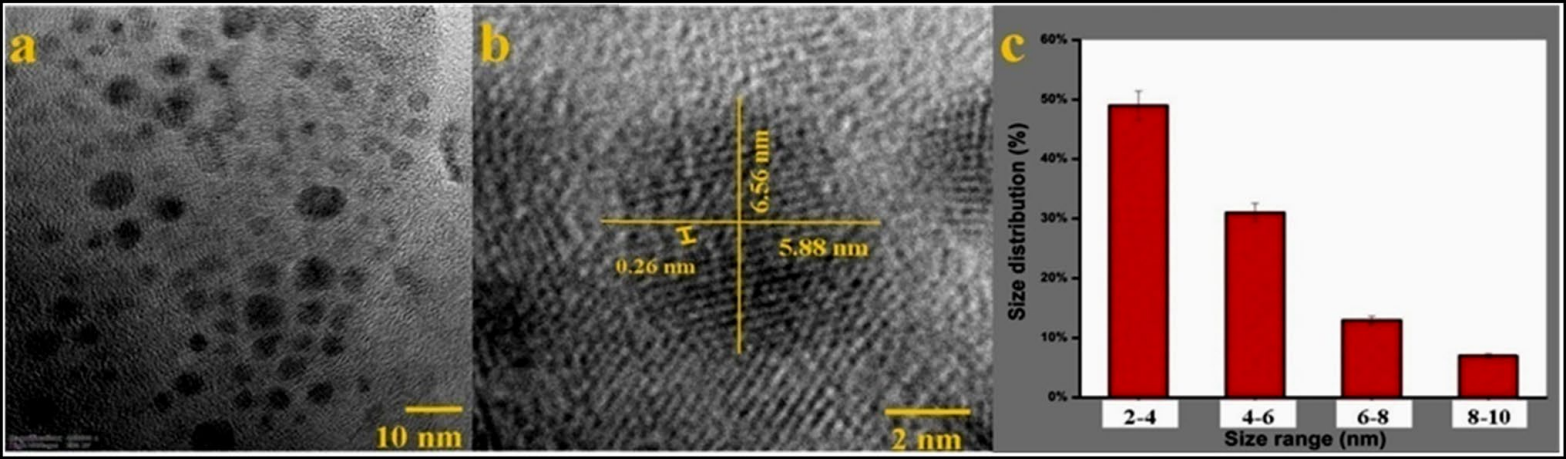
TEM images of ZnO NPs at **a** low and **b** high magnification and **c** size distribution graph of the NPs

**Fig. 5:**
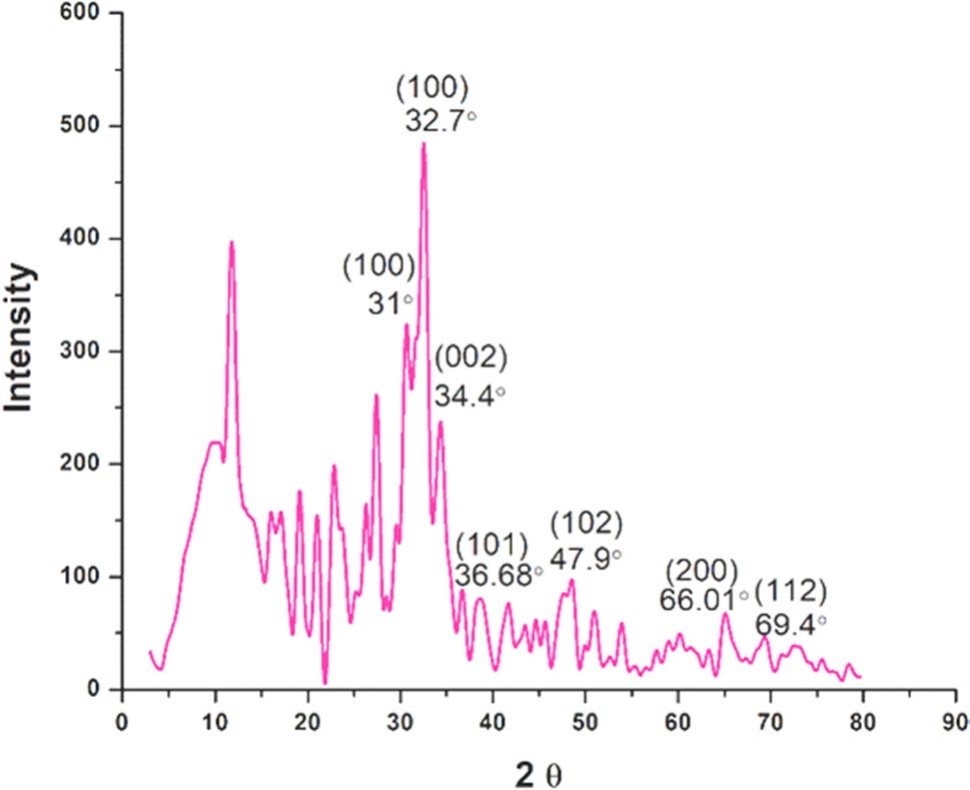
The peaks obtained from XRD spectra showing the fcc lattice points in the ZnO NPs

The synthesis of ZnO NPs was confirmed by the appearance of a white precipitate formed due to the reduction of ZnCl_2_ with biosurfactant after autoclaving. Scanning electron microscopic (SEM) analysis confirmed the successful formation of ZnO NPs with uniform morphology (Fig. 3a, b), whereas EDX analysis confirms the presence of metallic ZnO NPs in the sample (Fig. 3c). The TEM analysis also shows well-dispersed ZnO NPs (Fig. 4a, b) with a size range of 2–10 nm (Fig. 4c). The HR-TEM image shows molecular fringes of 0.26 nm (Fig. 4b) in the single crystal ZnO NPs and approximately 50% of the overall NPs exhibited the size range of 2–4 nm (Fig. 4c). The XRD results of ZnO NPs show the presence of prominent peaks (*2θ* values) at 31°, 32.7°, 34.4°, 36.68°, 47.9°, 66.01° and 69.4° which corresponds to (100), (100), (002),(101), (102), (200) and (112) planes of zinc oxide respectively which also confirm its crystalline nature (Fig. 5) [47–49]. The average crystal size of ZnO NPs was determined as 17.2 ± 0.09 nm based on Scherrer formula using the XRD data.

**Fig. 6:**
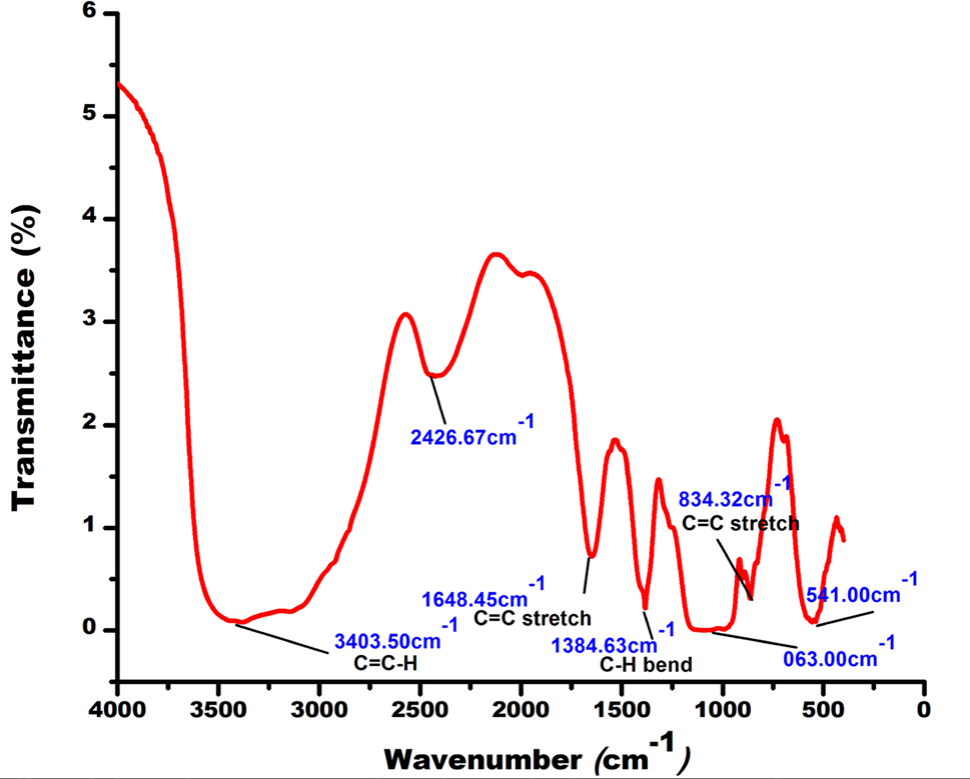
FTIR spectra of ZnO NPs indicating the functional groups present in the capping agent

**Fig. 7:**
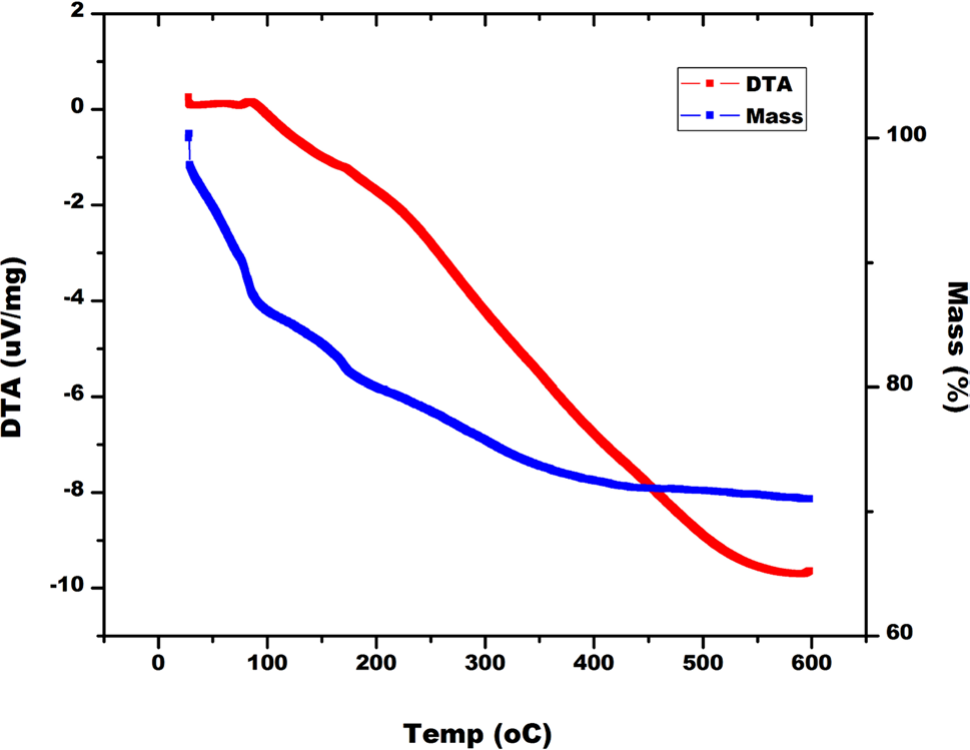
DTA-TGA graph of ZnONPs showing the gravimetric loss with respect to the change in temperature

The FTIR spectra of ZnO NPs showed distinct peaks in the regions 3403.50, 1648.45, 1384.63, and 834.32 cm^−1^. The peaks have confirmed the presence of C = C–H, C = C (stretch), C–H (bend), and C = C (stretch) respectively in ZnO NPs (Fig. 6) as they were synthesized by reducing the metal salt with bacterial biosurfactant [49]. The thermogravimetric analysis (TGA) and differential thermal analysis (DTA) spectra of ZnO NPs indicate that the NPs lose mass at temperatures above 100 °C (Fig. 7). Weight loss is minimal at temperatures below 100 ºC and above 500 ºC (Fig. 7). The DTA patterns exhibit a distinct maximum at 100 ºC, which aligns with the findings of the TGA (Fig. 7).

**Fig. 8:**
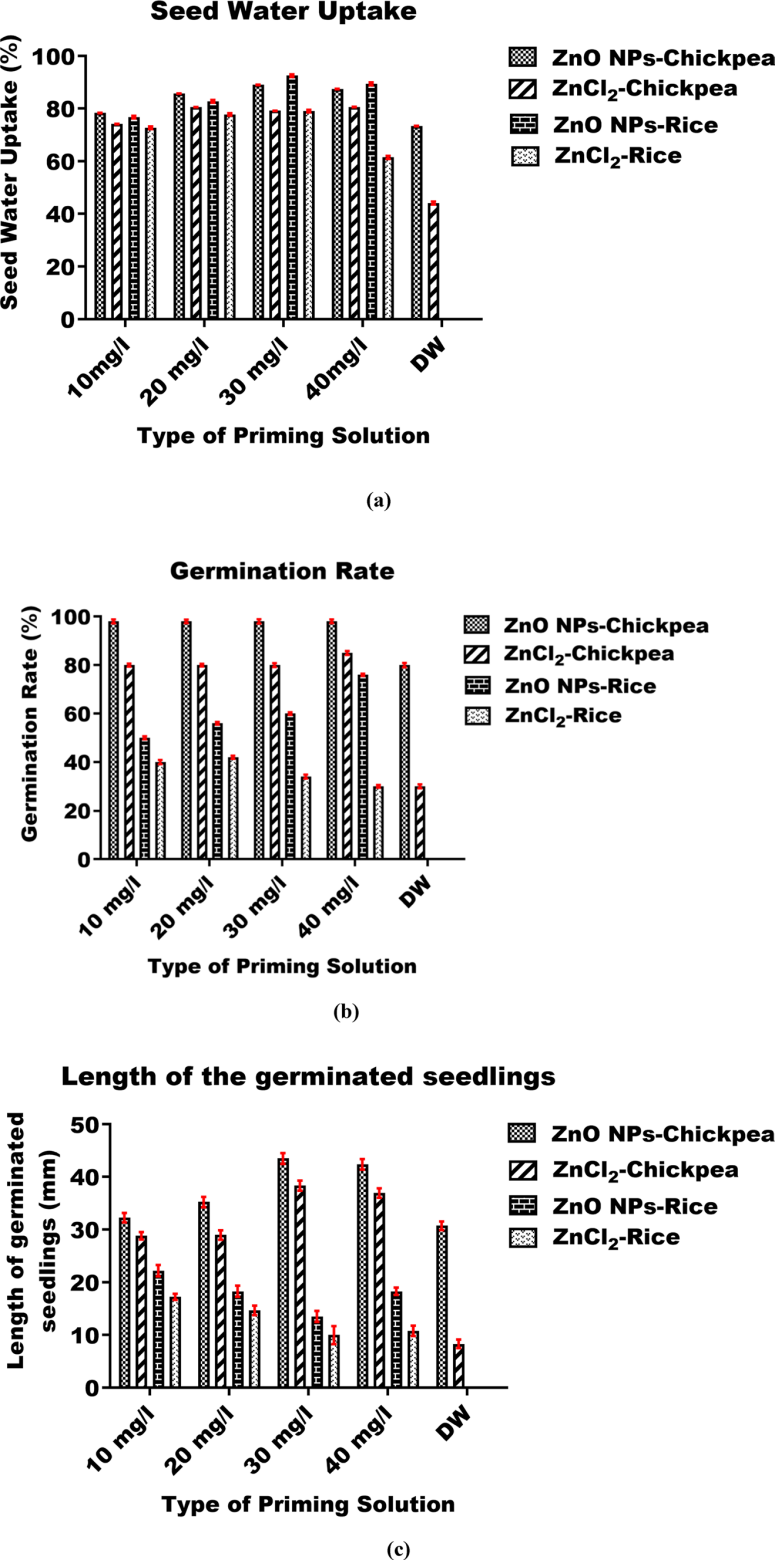
Graph shows the result of **a** seed water uptake, **b** germination rate, and **c** length of the germinated seeds of chickpea (*Cicer arietinum*), and rice (*Oryza sativa*) seeds after priming with various concentrations of ZnO NPs. Distilled water (DW) was used as control to see its effect on chickpea and rice seeds. All the values are expressed in terms of mean ± S.D

The percentage of water uptake was found higher in chickpea and rice seeds when primed with ZnO NPs as compared to distilled water, and ZnCl_2_ primed seeds. The water absorption capacity of both the seeds increased with the rise in the concentration of ZnO NPs priming solution. The seed water uptake percentage of primed chickpea seeds at 10, 20, 30, and 40 mg/L of ZnO NPs dosage was obtained as 78.37 ± 0.07%, 85.77 ± 0.06%, 89.06 ± 0.06%, and 87.49 ± 0.07% respectively which was found significantly higher (*p* ≤ 0.05) as compared to distilled water (73.47 ± 0.06%), and different concentration of ZnCl_2_ (10 mg/L–74.21 ± 0.06%, 20 mg/L–80.57 ± 0.06%, 30 mg/L–79.17 ± 0.05%, and 40 mg/L–80.62 ± 0.06%) (Fig. 8a). The percentage of water uptake in ZnO NPs primed chickpea seeds increases with the rise in the concentration from 10 to 30 mg/L and gradually reduces at 40 mg/L of nano-primed seeds which is shown in Fig. 8a. The maximum water uptake achieved was 89.06 ± 0.06% at 30 mg/L of ZnO NPs primed chickpea seeds. Similarly, the seed water uptake percentage of ZnO NPs primed rice seeds at 10, 20, 30, and 40 mg/L dosage was 76.8 ± 0.5%, 82.8 ± 0.5%, 92.54 ± 0.5%, and 89.39 ± 0.55% respectively which was found significantly higher (*p* ≤ 0.05) as compared to distilled water (44.1 ± 0.58%), and different concentration of ZnCl_2_ (10 mg/L–72.8 ± 0.5%, 20 mg/L–77.83 ± 0.45%, 30 mg/L–79.11 ± 0.47%, and 40 mg/L–61.52 ± 0.57%) treated seeds (Fig. 8a). As the concentration of ZnO NPs increases from 10 to 30 mg/L, the rate of water uptake also increases but gradually declines at 40 mg/L of ZnO NPs priming solution (Fig. 8a). The maximum water uptake was 92.54 ± 0.5% at 30 mg/L priming concentrations of ZnO NPs for rice seeds (Fig. 8a).

The germination percentage of ZnO NPs primed chickpea and rice seeds were found higher when compared with that of the control. The percentage of germination in chickpea seeds with 10, 20, 30, and 40 mg/L of ZnO NPs dosage was found 98 ± 0.8% which was significantly higher (*p* ≤ 0.05) than that of the germination percentage achieved by priming with distilled water (80 ± 0.9%), and different concentration of ZnCl_2_ (10 to 30 mg/L–80 ± 0.7%, and 40 mg/L–85 ± 0.8%). The percentage of germination in chickpea seeds remains constantly high at all dosages of ZnO NPs priming as represented in Fig. 8b. The maximum germination percentage achieved was 98 ± 0.8% and it remained the same for all the concentrations of ZnO NPs for chickpea seeds (Fig. 8b). The growth of the seedlings with 10, 20, 30, and 40 mg/L of ZnO NPs dosage was achieved as 32.3 ± 0.9, 35.25 ± 0.99, 43.5 ± 1.03, and 42.4 ± 1 mm respectively which was higher as compared to distilled water (30.75 ± 0.8 mm), and different concentration of ZnCl_2_ (10 mg/L–28.85 ± 0.7 mm, 20 mg/L–29 ± 0.88 mm, 30 mg/L–38.35 ± 0.96 mm, and 40 mg/L–36.95 ± 0.9 mm) primed seeds (Fig. 8c). The maximum average length achieved was 43.5 ± 1.03 mm at 30 mg/L concentration of ZnO NPs on chickpea seedlings, beyond which no major changes in the growth of seedlings were observed at 40 mg/L concentration of ZnO NPs primed seeds (Fig. 8c).

**Fig. 9:**
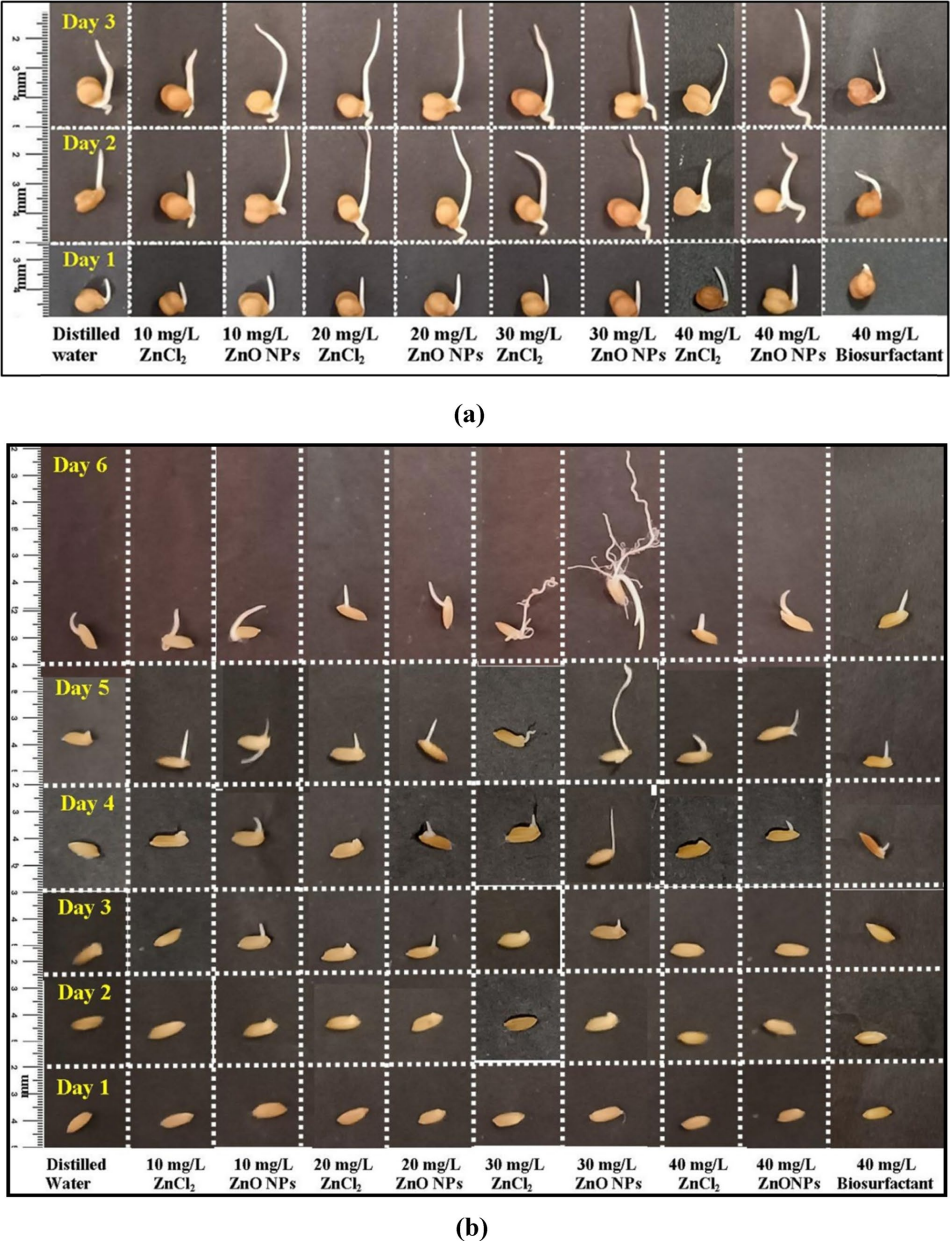
Overall growth profile of **a** chickpea seeds after ZnO NPs priming, and **b** rice seeds after ZnO NPs priming. Where distilled water and biosurfactant primed seeds are kept as control

The percentage of germination in ZnO NPs primed rice seeds with 10, 20, 30, and 40 mg/L of dosage was found 50 ± 0.6, 56 ± 0.55, 60 ± 0.54, and 76 ± 0.45% respectively which is significantly higher (*p* ≤ 0.05) as compared to the germination percentage achieved with distilled water (30 ± 0.76%), and different concentration of ZnCl_2_ (10 mg/L–40 ± 0.88%, 20 mg/L–42 ± 0.66%, 30 mg/L–34 ± 0.76%, and 40 mg/L–30 ± 0.55%) primed seeds after the same duration of time. The percentage of germination was found to increase with the rise in concentration from 10 to 40 mg/L and maximum germination percentage was observed at 40 mg/L of ZnO NPs priming dose (76 ± 0.45%) (Fig. 8b). The growth of the seedlings with 10, 20, 30, and 40 mg/L of ZnO NPs dosage was found 22.22 ± 1.08, 18.33 ± 1.07, 13.57 ± 1.04, and 18.33 ± 0.7 mm respectively which was found significantly higher (*p* < 0.05) as compared to distilled water (8.33 ± 0.81 mm), and different concentration of ZnCl_2_ (10 mg/L–17.27 ± 0.6, 20 mg/L–14.7 ± 0.9, 30 mg/L–10 ± 1.73, and 40 mg/L–10.83 ± 1 mm) primed seeds (Fig. 8c). The maximum average growth of the seedlings was observed as 22.22 ± 1.08 mm at 10 mg/L concentration of ZnO NPs on rice seedlings and no significant increase in length was observed at 20–40 mg/L concentration on rice seeds (Fig. 8c). The overall growth profile of chickpea and rice seeds at different time interval under influence of priming solution is depicted in Fig. 9a and b.

**Fig. 10:**
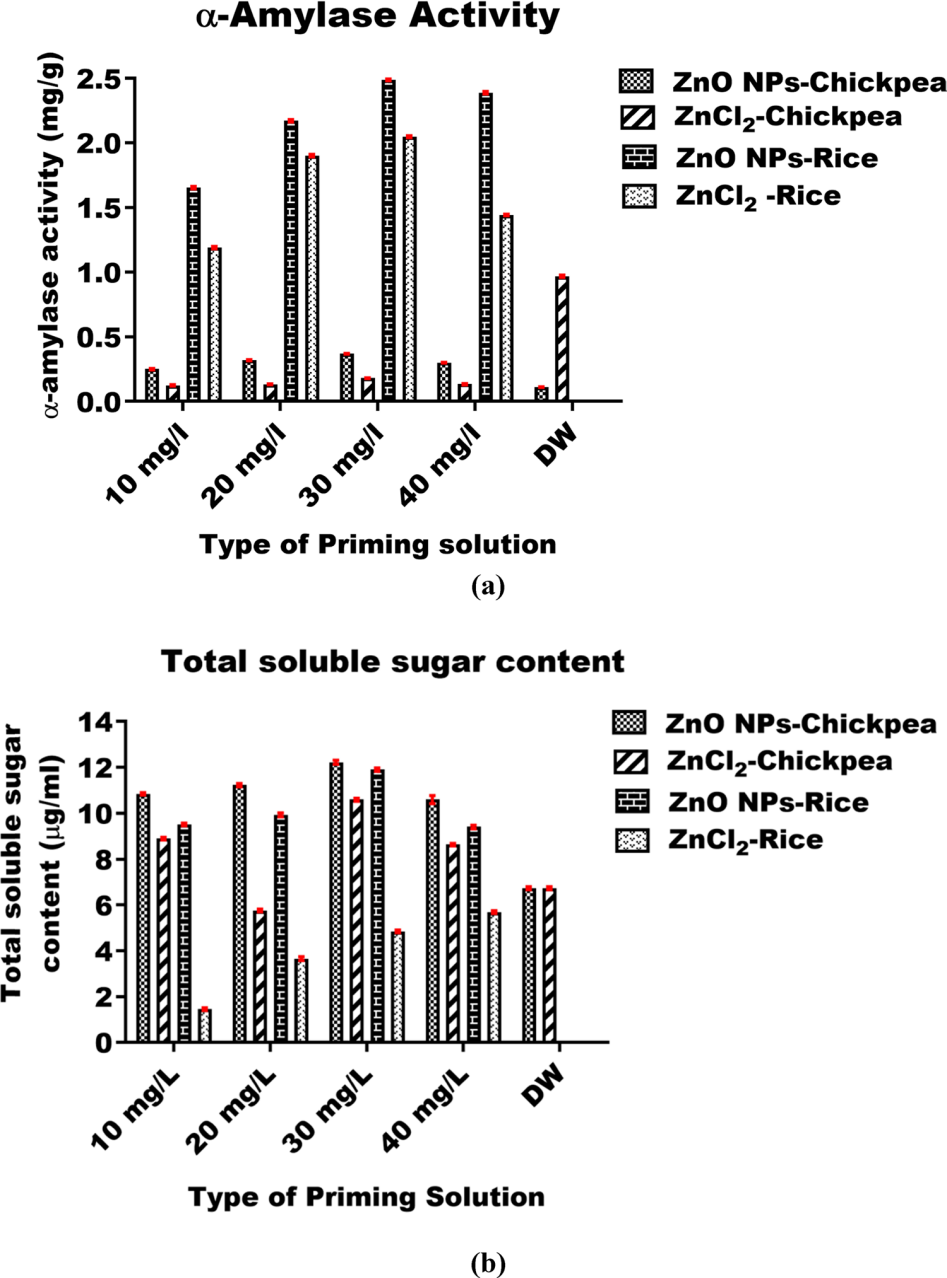
Graph shows **a** alpha amylase activity profile, and **b** total soluble sugar profile of the treated seeds. Distilled water (DW) was used as control to see its effect on chickpea and rice seeds. All the values are expressed in terms of mean ± S.D

The α-amylase activity in ZnO NPs primed chickpea seeds at 10, 20, 30, and 40 mg/L of dosage was found 0.25 ± 0.0048, 0.32 ± 0.0048, 0.37 ± 0.0024, and 0.3 ± 0.0037 mg/g respectively which was found significantly higher (*p* ≤ 0.05) as compared to distilled water (0.11 ± 0.0028 mg/g), and different concentration of ZnCl_2_ (10 mg/L–0.12 ± 0.0048 mg/g, 20 mg/L–0.13 ± 0.0028 mg/g, 30 mg/L–0.18 ± 0.0024 mg/g, and 40 mg/L–0.13 ± 0.005 mg/g) primed seeds (Fig. 10a). As the concentration of ZnO NPs dosage increases from 10 to 30 mg/L, the α-amylase activity also increases (Fig. 10a). However, no further significant enhancement in enzyme activity was observed at 40 mg/mL dose of ZnO NPs on chickpea (Fig. 10a). The maximum α-amylase activity was determined as 0.37 ± 0.0024 mg/g at 30 mg/L priming concentrations of ZnO NPs for chickpea seeds (Fig. 10a). Similarly, the α-amylase activity of ZnO NPs primed rice seeds at 10, 20, 30, and 40 mg/L concentration was found 1.65 ± 0.0097, 2.17 ± 0.009, 2.49 ± 0.0064, and 2.39 ± 0.0084 mg/g respectively which was found significantly higher (*p* ≤ 0.05) as a comparison to distilled water (0.97 ± 0.00843 mg/g), and different concentration of ZnCl_2_ (10 mg/L–1.2 ± 0.0097 mg/g, 20 mg/L–1.9 ± 0.0097 mg/g, 30 mg/L–2.05 ± 0.0073 mg/g, and 40 mg/L–1.44 ± 0.0055 mg/g) primed seeds (Fig. 10a). The increase in the concentration of ZnO NPs from 10 to 30 mg/L enhances the α-amylase activity but no significant improvement in the enzymatic activity was observed at 40 mg/L of ZnO NPs priming solution (Fig. 10a). The maximum α-amylase activity was recorded as 2.49 ± 0.0063 mg/g at 30 mg/L priming concentrations of ZnO NPs in rice seeds.

The concentration of soluble sugar of ZnO NPs primed chickpea seeds at 10, 20, 30, and 40 mg/L dosage was found 10.85 ± 0.05, 11.22 ± 0.07, 12.23 ± 0.087, and 10.6 ± 0.18 µg/mL respectively which was found significantly high (*p* ≤ 0.05) as compared to distilled water (6.73 ± 0.06 µg/mL), and different concentration of ZnCl_2_ (10 mg/L–8.9 ± 0.057 µg/mL, 20 mg/L–5.76 ± 0.058, 30 mg/L–10.6 ± 0.06 µg/mL, and 40 mg/L–8.64 ± 0.051 µg/mL) primed seeds. As the concentration of ZnO NPs dosage increases from 10 to 30 mg/L, the amount of sugar also increases but again decreases at 40 mg/mL concentration of the primed solution in chickpea seeds (Fig. 10b). The highest sugar content was determined as 12.23 ± 0.08 µg/mL at 30 mg/L priming concentration of ZnO NPs in chickpea seeds. Similarly, the sugar content of ZnO NPs primed rice seeds at 10, 20, 30, and 40 mg/L dosage was found 9.52 ± 0.07, 9.94 ± 0.08, 11.9 ± 0.07, and 9.4 ± 0.07 µg/mL respectively and was found significantly high (*p* < 0.05) as compared to distilled water (6.73 ± 0.06 µg/mL), and different concentration of ZnCl_2_ (10 mg/L–1.45 ± 0.06 µg/mL, 20 mg/L–3.66 ± 0.09 µg/mL, 30 mg/L–4.84 ± 0.06 µg/mL, and 40 mg/L–5.69 ± 0.06 µg/mL) primed seeds. The increase in the concentration of ZnO NPs from 10 to 30 mg/L enhanced the amount of total soluble sugar content in seeds but decreased at 40 mg/L concentration of ZnO NPs priming solution (Fig. 10b). The maximum soluble sugar content was found 11.9 ± 0.07 µg/mL at 30 mg/L of priming concentrations for rice seeds. The overall growth of chickpea and rice seedlings after treating with the priming solution at different time interval is depicted in Fig. 10b.

**Fig. 11:**
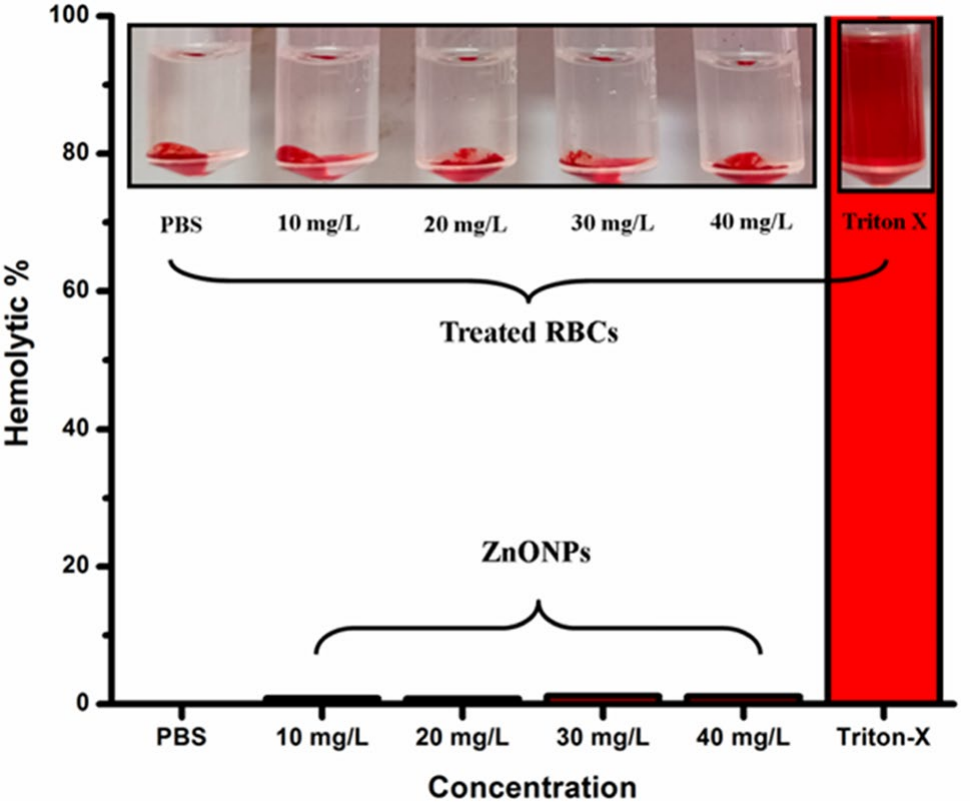
Cytotoxicity assessment of various concentrations of ZnO NPs on red blood cells. Where, PBS and Triton-X were used as negative and positive control respectively

The potential cytotoxicity of ZnONPs was assessed on red blood cells and shown to be non-hemolytic, as shown in Fig. 11. The addition of ZnO NPs at dosages of 10, 20, 30, and 40 mg/L resulted in hemolytic activity of 0.85 ± 0.005%, 0.8 ± 0.001%, 1.17 ± 0.002%, and 1.13 ± 0.002% correspondingly.

**Fig. 12:**
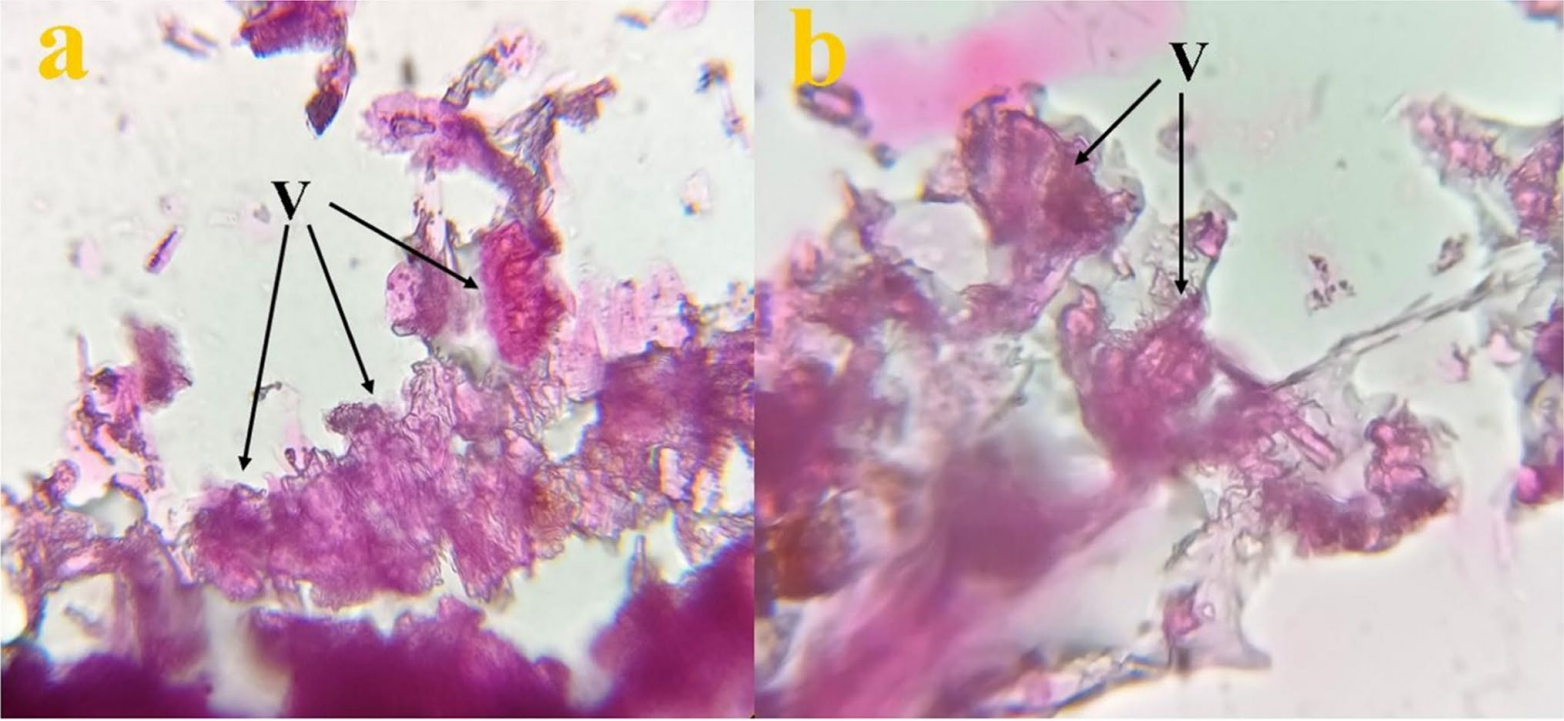
Histological staining of the **a** control [27] and **b** test (40 mg/L of ZnO NPs) shows healthy gut of earthworm (*Eudrilus eugeniae*) with healthy villi (V)

The earthworms subjected to treatment in both the control and test groups exhibited no mortality, no alteration in color, and no changes in behavior which showed no ecotoxic nature of the ZnO NPs (Table 2). The histological staining result of both the control [27] (Fig. 12a) and test (Fig. 12b) shows the presence of healthy villi (V) in the gut tissues of the earthworms. In addition to this, the treated earthworms also showed normal reproduction with offspring even after treatment with the NPs.

**Table 2:** Results of toxicity assessment on earthworms (*Eudrilus eugeniae*) at different concentration of ZnO NPs

Sl. No	Parameter analysed	Control (DW)	ZnONPs			
			10 mg/L	20 mg/L	30 mg/L	40 mg/L
1	Colour change	No	No	No	No	No
2	Behavioural change	No	No	No	No	No
3	Death	No	No	No	No	No

## Discussion

The agricultural sector plays a critical role in increasing food availability and ensuring food security [50, 51]. While there is widespread consensus that global food demand will increase in the future, there is uncertainty about whether global agriculture can meet this need through food supply expansion. Improving food provision through increased agricultural production and expanded agricultural land use appears to be a viable approach to eradicating hunger [51]. Throughout human history, significant technological innovations have been applied to enhance agricultural production despite limited resources. However, the combination of a growing population and climate change consistently presents challenges in balancing the availability and demand for food. It is projected that the global population will reach 9 billion by 2050, representing a 25% increase from the current population [52]. This population increase will be more significant in growing nations like Mexico, India, China, and other countries. Additionally, urbanization in developing nations is expected to rise by 2050 [1]. As living standards improve in the future, there will be a further surge in food demand, especially in developing countries [53].

In modern times, farmers and growers dedicate 70% of their time to monitoring and understanding the state of their crops rather than engaging in physical agricultural work. Thus, the agricultural sector requires the use of precise and advanced technologies to demonstrate advancements in farming [54].

Growing industrialization and the increase in carbon emissions contribute significantly to soil quality degradation and a decline in soil fertility [55]. These changes directly impact the quality and quantity of agricultural products [55].Chemical fertilizers are often regarded as a significant solution for addressing nutrient depletion and ensuring the continuity of food production [56]. However, the persistent use of synthetic fertilizers causes significant issues, such as soil nutrient depletion, food contamination, and damage to soil physicochemical properties. This damage is caused by an increase in soil acidity, which results in rapid deterioration of soil health, productivity, stability, and sustainability. When applied in the field, chemical fertilizers may temporarily remain in the soil or be lost through volatilization or leaching into groundwater, leading to environmental pollution [57]. Therefore, current agricultural trends highlight the pursuit of alternatives to non-renewable chemical fertilizers, primarily due to their high costs and the resulting environmental pollution. There is an urgent need for an efficient method to develop an environmentally friendly, affordable, and effective organic bio-fertilizer [57]. While the use of green nanotechnology in agriculture is mostly theoretical, a limited number of studies document the use of NPs to enhance productivity [27, 58]. Consequently, it is imperative for farmers to address theoretical concepts promptly to fully leverage the potential of nanotechnology in agriculture.

In the past ten years, there has been a dramatic rise in the demand for nano-sized materials, driven by the vision to develop innovative technologies. This enthusiasm is accompanied by a growing need to find environmentally friendly techniques for producing these materials. Biosurfactants have emerged as a more sustainable approach for manufacturing nano-sized materials. They serve as capping and reducing agents in NPs synthesis, contribute to the formation of self-assembly structures for encapsulation, functionalization, or templates, and act as emulsifiers in nano-emulsions [59].

In recent times, there has been a significant focus on ZnO NPs because of their ability to improve the nutrient uptake, seed germination, and the development and productivity of plants and crops. In addition, ZnO NPs have been linked to enhance the tolerance of abiotic stress in plants, including temperature stress, drought, heavy metal stress, and salt. This is achieved by enhancing the levels of antioxidant enzymes and metabolites [60]. Furthermore, zinc (Zn) is an essential micronutrient necessary for the proper growth and development of plants. Zinc not only plays a role in the production of proteins and metabolism of biomolecules, but it also promotes resistance to environmental challenges [60, 61]. Studies have reported the synthesis of ZnO NPs through green and chemical methods for agricultural applications, enhancing the seed germination of crops such as rice, wheat, maize, sorghum, and chickpeas [62–64]. However, these studies have primarily demonstrated the synthesis of ZnO NPs using plant parts, which may pose a threat to biodiversity if scaled commercially. Moreover, none have explored the potential of bacterial biosurfactant-mediated ZnO NPs for similar roles, including their mode of action and environmental toxicity.

*Klebsiella* sp. is a common Gram-negative, rod-shaped soil bacterium [65]. Various species of *Klebsiella* are known for their ability to produce biosurfactants and their application in bioremediation of complex petroleum hydrocarbons [66, 67]. Furthermore, these surfactants are known for maintaining stability even under severe and challenging environmental conditions, making them very valuable for commercial purposes [62, 66]. However, their ability to produce NPs for agricultural applications has been rarely explored. Recently, biosurfactant-producing *Klebsiella* sp. RGUDBI03 was reported for its ability to produce Ag NPs by reducing silver salt into metallic NPs [27]. This strain was further studied for its application in seed germination assays.

The drop collapse test conducted on the cell-free extract of the isolate showed that the drop rapidly lost its surface tension and fell entirely within 45 s. This phenomenon is attributed to the biosurfactant produced by the isolate in the medium supplemented with diesel [68–70]. The emulsification index (*E*_*24*_) is a significant parameter used to assess and describe bacteria that produce biosurfactants [71]. The isolate exhibiting the formation of an emulsion layer is regarded as a promising candidate for biosurfactant production [71]. This investigation focuses on *Klebsiella* sp. RGUDBI03, which exhibited an *E*_*24*_ value of 35.71% after 24 h against diesel, consistent with results reported by other studies [38, 71–73].

The FTIR spectrum analysis of the biosurfactant, as reported earlier, verified the existence of chemical groups such as O–H (bend), O–H (stretch), CH_2_ (asymmetrical stretch), C = C (stretch), C–H (bend), C–H in-plane bend, and C = C–H (bends), which represent the basic chemical characteristics of bacterial biosurfactants [27, 74–76].

The production of biosurfactant-mediated ZnONPs was first verified by the formation of a white solid precipitate [77]. The scanning electron microscopy (SEM) study confirmed the effective creation of ZnO NPs. Furthermore, the EDX analysis revealed distinct peaks that confirmed the presence of ZnO NPs, with maximum peaks correlating with the presence of zinc and oxygen in the tested sample. The energy-dispersive X-ray (EDX) spectra of the ZnONPs sample exhibited distinct peaks corresponding to zinc and oxygen, with energy levels around 1 eV and 9 eV, respectively [78]. These results showed that the reaction product consisted of ZnO NPs with a certain level of purity. Transmission electron microscopy (TEM) revealed the creation of distinct and evenly distributed NPs with an average size ranging from 2 to 10 nm. The XRD peaks were aligned using a typical JCPDS card no: 36–1451, indicating the existence of (100), (100), (002), (101), (102), (200), and (112) lattice points in the face-centered cubic crystal structure of ZnONPs. Similar findings have been documented in other scholarly sources [47–49]. The prominent Bragg peaks may be attributed to the biosurfactant, which functions as a capping agent and provides stability to the NPs [79]. The inclusion of organic chemicals used during the manufacture of ZnO NPs may account for some supplementary peaks [79]. The FTIR spectra of the biosynthesized ZnO NPs showed the presence of C = C–H, C = C (stretch), C–H (bend), and C = C (stretch). Both samples exhibited comparable peak intensities for the biosurfactant, confirming its favorable contribution to the synthesis of the NPs.

The sample's thermal stability and mass loss were assessed using TGA and DTA analysis. The results indicated that the sample undergoes thermal dissociation, with a gravimetric loss due to the evaporation of water and organic compounds occurring above 100 °C. This suggests that the sample has a durable stability profile and can withstand high temperatures [80, 81]. The DTA-TGA analytical findings of the ZnO NPs sample indicated a consistent decrease in weight above 100 °C, with little reduction in weight when heated over 500 °C.

**Fig. 13:**
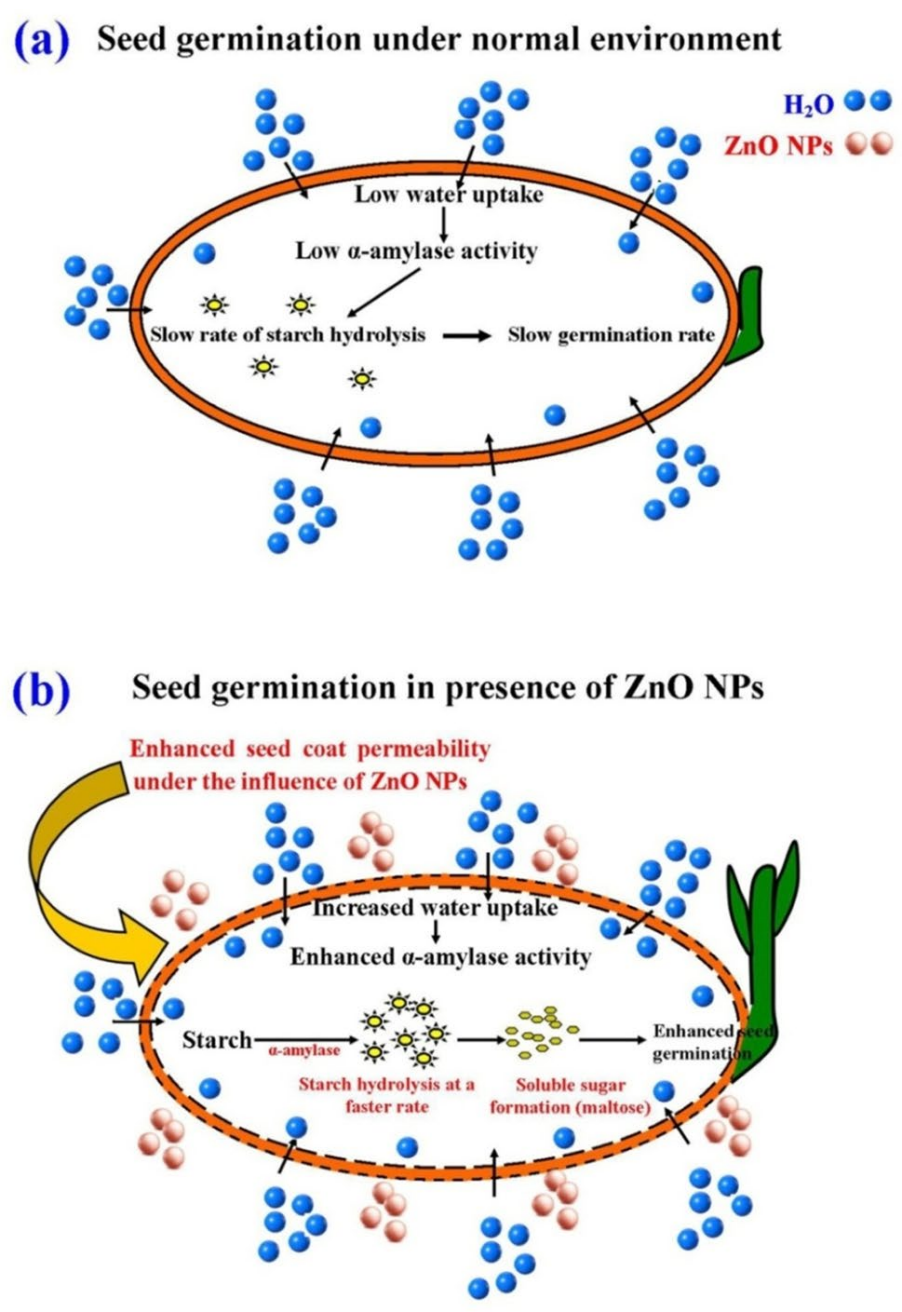
Illustration shows **a** seed germination under normal condition which involves uptake of water by plant seed leading to activation of α-amylase activities followed by the hydrolysis of starch at a slower rate and seed germination (adapted from Mahakham et al. (2017) [86] under Creative Commons Attribution 4.0 International License). Figure **b** shows the possible mode of action of ZnO NPs increasing the seed permeability leading to rapid absorption water followed by faster α-amylase activity and enhanced seed germination

Recent studies have shown that nano-priming is more effective than traditional priming methods in attaining significant agricultural yields. It involves the use of NPs that have a size smaller than 100 nm which improves the ability to withstand moderate and prolonged stress [82]. The underlying process of nano-priming and its ability to enhance seed germination is not yet well understood [83]. Nano-priming may alter the seed’s metabolism, resulting in enhanced water absorption, rapid breakdown of starch, loosening of cell walls, weakening of the endosperm, fast expansion of the embryo, and rapid development of the root and shoot. In addition, seed nano-priming may regulate several physiological and biochemical processes, including modifying the activity of the defense system and increasing levels of antioxidants and enzyme activities. This enhances the vigor of plants and improves their ability to withstand environmental obstacles [84]. Seeds treated with ZnONPs exhibited maximum water absorption, reaching around 89% and 93% for chickpea and rice seeds, respectively, when exposed to ZnO NPs at a concentration of 30 mg/L. Furthermore, it was noted that the concentration of soluble sugars was higher in seeds treated with NPs compared to untreated seeds. This increase in soluble sugar content was directly proportional to the dosage of ZnONPs. The likely cause for the rise in water absorption might be attributed to the osmotic difference inside and outside the tissues [85]. Based on the results of this study, it is hypothesized that priming seeds with ZnO NPs enhances their ability to absorb water. This, in turn, may accelerate the enzymatic activity of α-amylase, a crucial enzyme for promoting seed germination [86–88]. Multiple pieces of evidence indicate a higher rate of starch breakdown in seeds treated with NPs, resulting in an enhanced rate of seed germination [89]. Several recent studies have shown that α-amylase activity is catalyzed, resulting in increased water absorption capacity in rice and chickpea seeds treated with NPs. This leads to enhanced starch breakdown and germination rate [70, 86, 90, 91]. Enhancing the activity of α-amylase promotes the breakdown of starch into maltose and other types of soluble sugars, leading to a progressive improvement in the development rate of the seedlings [86, 92]. Based on these findings supported by existing literature, the proposed mode of action of ZnO NPs in enhanced seed germination is illustrated in Fig. 13.

The biosurfactant-mediated ZnO NPs was further evaluated for toxicity assessment on red blood cells and earthworms to ensure the safe commercialization of the product in future. Sheep blood cells did not show any indication of hemolysis when exposed to concentrations of ZnO NPs ranging from 10 to 40 mg/L. The results were compared with the positive control (Triton X), which demonstrated 100% hemolysis, and the negative control (PBS), which showed no hemolysis. Substances that exhibit hemolytic activity of less than 2% are often regarded as non-cytotoxic [93]. Thus, the results prove that ZnO NPs are non-cytotoxic in nature and thus possess no harm to living organisms [45, 94].

Soil contamination has become a significant global issue in recent decades. Assessment of soil contamination is an essential strategy for preserving living beings and the environment. The conventional physicochemical method used to evaluate the threat of soil pollution relies on measuring the concentration of pollutants in the soil. However, this technique is insufficient and does not provide information on the harmful impact of the pollutants on living organisms [95]. Earthworms are widely recognized as viable bio-monitoring organisms for assessing soil contaminants [96, 97]. They are often used as a bio-indicator for soil health due to their sensitivity to harmful substances and other distinctive biological benefits. Some international organizations such as ISO (International Organization for Standardization) and OECD (Organization for Economic Co-operation and Development) have recognized earthworms as appropriate model species or standard test organisms in soil ecotoxicology studies [95, 98]. The absence of any alteration in earthworm activity indicates that the produced ZnO NPs are environmentally and soil-friendly. The earthworms' survival rate when exposed to chemicals provides a concise indication of the environmental safety evaluation of any substance [27, 99].

In this work, earthworms (*Eudrilus eugeniae*) were exposed to ZnO NPs at a maximum concentration of 40 mg/L, which is often used for seed germination assays. Distilled water was utilized as a control treatment. After 6 days of treatment, the observation revealed no discernible physiological, behavioral, or color alterations in the earthworms. Furthermore, examination of gut tissues using histological staining revealed a healthy gut with villi (V), supporting future field studies. The observation of an intact epithelial layer with intestinal villi in the treated earthworms signifies healthy gut tissues and demonstrates the non-ecotoxic nature of ZnO NPs [100].

However, long-term exposure to high concentrations of ZnO nanoparticles (NPs) can negatively affect soil health and microbial communities. At relatively low concentrations, ZnO NPs have been reported to stimulate the morphometric and biochemical properties of plants, as well as enhance soil microbiota and its activity [101, 102]. Conversely, when ZnO NP concentrations exceed certain thresholds, hazardous effects may occur. The impact of ZnO NPs is influenced by factors such as particle size, dosage, duration of exposure, water solubility, soil composition, acidity, and organic matter content [103–105]. To develop nano-agrochemicals for improved seed treatment, future research should consider these factors carefully. Optimizing agrochemical parameters at the nanoscale is crucial to achieve consistent and beneficial results from seed treatments. Determining the optimal dose and ensuring its precise delivery are key to enhancing treatment efficacy [106]. The findings indicate that biosynthesized ZnO NPs, at low concentrations, demonstrated superior effectiveness in promoting seed germination, highlighting their potential for future field trials.

## Conclusions

A drastic revolution in the field of agriculture to improve crop productivity is urgently needed, and nanotechnology may cater to the agricultural sector with its gradual intervention. This study reports the synthesis of stable ZnO NPs with the help of bacterial biosurfactant, instead of plant or animal extract, thereby minimizing the chances of hampering biodiversity. These ZnO NPs were tested for their ability to enhance the seed germination rate of rice and chickpea, which are of great commercial value. A steep increase in water uptake by the ZnO NPs-primed seeds was observed, elevating the α-amylase activity and enhancing seed germination. Further, healthy villi exhibited by the gut of the ZnO NPs-treated earthworms advocated its non-ecotoxic nature and the futuristic potential for field implementation. Additionally, the ZnO NPs also showed no cytotoxicity on RBC cells, justifying its safe use for future studies.

## Data Availability

The partial sequence of the 16S ribosomal RNA gene of Klebsiella sp. strain RGUDBI03 presented the manuscript is available of NCBI GenBank database with the accession no. ON945613.1 and may be accessed by following the link https://www.ncbi.nlm.nih.gov/nuccore/ON945613.1. All the data related to the characterization of the biosurfactant may be accessed from the research paper Das et al., (2024) (https://doi.org/10.1007/s12668-024–01444-7).
